# Plants clonal strategies are well associated with aridity gradients: insights from Lamiaceae family in the SW and Central Asia

**DOI:** 10.1093/aobpla/plaf069

**Published:** 2025-12-08

**Authors:** Chonour Mahmoudi, Jalil Noroozi, Massoud Ranjbar, Matěj Man, Sina Khalvati, Jitka Klimešová, Tomáš Herben

**Affiliations:** Department of Botany, Faculty of Science, Charles University, Benátská 2, Praha 128 01, Czech Republic; Department of Botany and Biodiversity Research, University of Vienna, Rennweg 14, Vienna A-1030, Austria; Department of Biology, Herbarium Division, Bu-Ali Sina University, Shahid Fahmideh Blvd, Hamedan 65178-38695, Iran; Department of Botany, Faculty of Science, Charles University, Benátská 2, Praha 128 01, Czech Republic; Department of Geoecology, Institute of Botany of the Czech Academy of Sciences, Zámek 1, Průhonice 252 43, Czech Republic; Department of Biology, Faculty of Sciences, Hakim Sabzevari University, Daneshjoo Boulevard, Sabzevar 96179-76487, Iran; Department of Botany, Faculty of Science, Charles University, Benátská 2, Praha 128 01, Czech Republic; Department of Experimental and Functional Morphology, Institute of Botany, Czech Academy of Sciences, Dukelská 13, Třeboň 379 82, Czech Republic; Department of Botany, Faculty of Science, Charles University, Benátská 2, Praha 128 01, Czech Republic; Department of Population Ecology, Institute of Botany of the Czech Academy of Sciences, Zámek 1, Průhonice 252 43, Czech Republic

**Keywords:** aridity gradients, belowground growth forms (BGFs), clonal growth organs (CGOs), clonal reproduction, generalized additive model (GAM), Lamiaceae family, SW and Central Asia, species distribution

## Abstract

Clonal reproduction is often considered advantageous in stressful environments. While considerable research has explored how clonality supports plant survival in wet and cold conditions, its role in arid and semi-arid conditions remains underexplored. To address this gap, this study examines the distribution and diversity of clonality as a key component of belowground growth form (BGF) along aridity gradients across SW and Central Asia using the species-rich Lamiaceae family as a model. Data were collected from 281 species with a variety of BGFs occurring in a broad range of habitats. Data on BGFs were collected primarily in the field, with additional data from herbarium records and digital databases. BGFs were categorized into hypogeogenous rhizomes, epigeogenous rhizomes, stolons, and non-clonal types. Species distribution data were obtained from regional floras and the Global Biodiversity Information Facility (GBIF) and analysed using precipitation-related bioclimatic variables. Clonal species of the Lamiaceae family, particularly those with hypogeogenous and epigeogenous rhizomes, were more prevalent in extreme environments, both water-limited and moisture-rich, highlighting their adaptation to stressful conditions. They thrived in arid habitats like deserts and semi-deserts as well as wet habitats such as forests or wetlands. Non-clonal species were concentrated in the centre of the gradient, dominating montane steppe shrublands where water availability was moderate and seasonally variable. Clonal plants are not avoiding arid environment. This is particularly noteworthy for species with hypogeogenous rhizomes that have been shown to prefer wet conditions in temperate regions. The exact mechanisms that permit their specialization to wet or dry conditions is to be further studied experimentally. These findings highlight how climate change may differentially affect species based on their BGFs.

## Teaser

Our study demonstrates that plant clonality is strongly structured by aridity gradients across Southwest and Central Asia. Using an extensive dataset of Lamiaceae species, we show that belowground growth forms are highly represented in distinct climatic niches, particularly in both the driest and the wettest environments. These results highlight how underground organs enable plants to persist under water-limited conditions and improve predictions of vegetation responses to future climate change.

## Introduction

Understanding the ecological strategies that enable plants to thrive in diverse environments is a cornerstone of ecological research, with clonal reproduction playing an important, albeit often overlooked, role in plant adaptation and survival, particularly under environmental stresses (Callaghan et al. 1992, Carlsson et al. 1992, Eckert et al. 2003, Klimešová and Doležal 2011). This form of asexual reproduction, which involves the formation of new individuals from specialized clonal (usually belowground) organs, provides distinct advantages in challenging environments where sexual reproduction is limited. Belowground morphology facilitates persistence by enabling the production of genetically identical offspring potentially well-adapted to local adverse conditions. Moreover, vegetative structures serve as storage organs and promote rapid spread, contributing to the dominance of perennial species across ecosystems such as grasslands, tundra, wetlands, and aquatic systems (Grace 1993, Klimeš et al. 1997, Klimešová at al. 2012, Barrett 2015). However, despite their ecological importance, belowground morphologies and clonal growth strategies were never studied in extremely arid conditions where their contribution to plant persistence and ecosystem functions may be crucial for ecosystem resilience (Rice 2002, Klimešová et al. 2023).

The advantages of clonal reproduction are further emphasized by the diverse morphological features that enable clonality, which are categorized into clonal growth organs (CGOs). Clonality is not a single trait but is expressed through a range of CGOs that differ in their structure, function, and ecological roles. These CGOs are defined based on three key characteristics: the bud-bearing organ (morphological origin, such as stem or root), its position relative to the soil surface, and its role as a storage organ (shoot, root, or leaf) (Klimešová et al. 2018, 2019). Among the CGOs, the most common types are organs of stem origin like stolons (aboveground horizontal rooting stems), epigeogenous rhizomes (developed at the soil surface with green leaves and later pulled belowground), and hypogeogenous rhizomes (formed beneath the soil surface with scale leaves only). Non-clonal perennial herbs do not possess any CGO; their specific morphology is characterized by perennial main root (taproot).

The morphological types of clonal and non-clonal organs differ in their ecological functions and their distribution along environmental gradients, with stolons often associated with rapid spread in open and disturbed environments, and hypogeogenous and epigeogenous rhizomes adapted to more stable but stressful habitats (Sosnová et al. 2010, Herben et al. 2014) The hypogeogenous rhizomes have been shown to prefer wet while epigeogenous rhizomes and non-clonal plants to prefer dry habitats (Klimešová and Herben 2024). While non-clonal plants with perennial main root can attain deep soil horizons that contain water even in a dry season, clonal plants, especially those with stolons, are characterized by shallower rooting but thanks to interconnected network of stems bearing roots, they may root over large area (Klimešová and Herben 2023). These differences may affect distribution of clonal and non-clonal morphologies along aridity gradients and indeed clonal plants are more often reported from wet in comparison with dry sites (Klimeš et al. 1997, Sosnová et al. 2010, Klimešová and Herben 2015).

Despite the recognized specialization of clonal and non-clonal plants along water-availability gradients, much of the research addressing large-scale patterns of clonality has focused on temperate European systems (Klimešová and Herben 2024). As a result, our understanding of clonality is heavily biased towards mesic and wet environments, and clonal strategies in arid and semiarid regions remain understudied. Recent regional and continental studies, however, show that patterns of clonality vary widely across the globe—for example, latitudinal and regional surveys have documented clonality gradients in Australia and East Asia (Zhang et al. 2018, Fujinuma et al. 2019) and a continental-scale analysis in China found increased clonality towards cold, dry, or very wet environments (Ye et al. 2014). Detailed local studies also indicate strong climatic control of clonal and bud-bank traits in forest understories (Chelli et al. 2019) and pronounced habitat associations of clonal growth forms in Arctic plant communities (Klimešová et al. 2012). Moreover, wetlands often host a disproportionately high share of clonal species that are monocots, complicating simple interpretations of clonality–water relationships across habitat types (Klimeš et al. 1997, Song and Ming 2002, Sosnová et al. 2010). Therefore, expanding research to encompass arid and semiarid regions—with explicit attention to individual CGOs in dicotyledonous taxa—is essential to capture the full spectrum of clonal adaptations and the ecological pressures that shape these reproductive strategies.

We studied the distribution of CGOs and nonclonal belowground organs in a region characterized by precipitation gradient in SW and Central Asia. This region, which spans from humid coastal areas to hyper-arid deserts, is characterized by some of the most extreme climatic conditions on Earth, with temperatures among the highest globally and available water resources falling below 20% of the global average. We worked with the eudicot family Lamiaceae as it has high number of species, but still manageable to study all of them in the field, it has high diversity of BGO, wide ecological distribution, and well-documented taxonomy and phylogeny. The presence of 64 genera and approximately 470–500 species within SW and Central Asia, combined with comprehensive distribution and taxonomic data (Azizi Jalilian et al. 2020, Noroozi et al. 2021, Fonseca et al. 2021, Zhao et al. 2021a, 2021b), underscores the significance of focusing on the Lamiaceae.

Following Klimešová and Herben (2024), we used the collective term belowground growth forms (BGFs) to characterize structure of belowground organs of the clonal and nonclonal species. By studying the distribution and diversity of BGFs within the Lamiaceae across the varied environments of SW and Central Asia, we aim to address following questions: How do the percentage of clonal species and the occurrence of individual CGO types associate with the (i) precipitation gradient and (ii) different habitat conditions (approximated by vegetation types)? We hypothesize that in wet environments and in communities with high water availability hypogeogenous rhizomes and stolons will prevail, while in arid climate and communities with low water availability nonclonal plants will be the most common BGF.

## Materials and methods

### Study area

The study area encompasses SW and Central Asia, specifically including Turkey, Armenia, Azerbaijan, Lebanon, Israel and Palestinian territories, Syria, Iraq, Saudi Arabia, Yemen, Oman, United Arab Emirates, Iran, Afghanistan, Pakistan, Tajikistan, Kyrgyzstan, and Uzbekistan. The climate within this region exhibits considerable variation, transitioning from arid and semi-arid conditions that dominate regions such as the Arabian Peninsula, the central areas of Iranian Plateau, and the eastern Afghanistan, to more temperate climates observed in regions including northern Turkey, the Levant (comprising Lebanon and Syria), and the Hyrcanian region in northern Iran (Zohary 1973, Al-Bakri et al. 2013, Noroozi et al. 2016, 2018, Noroozi 2020). The average annual precipitation ranges from less than 100 mm in the driest areas to over 1000 mm in the wettest, with temperature variations spanning from below freezing in winter to above 50°C in summer (Zittis et al. 2016). These climatic extremes offer a unique opportunity to investigate how plant species adapt to such contrasting environments through clonal growth strategies.

### Field work and specimen collection

**Figure 1:**
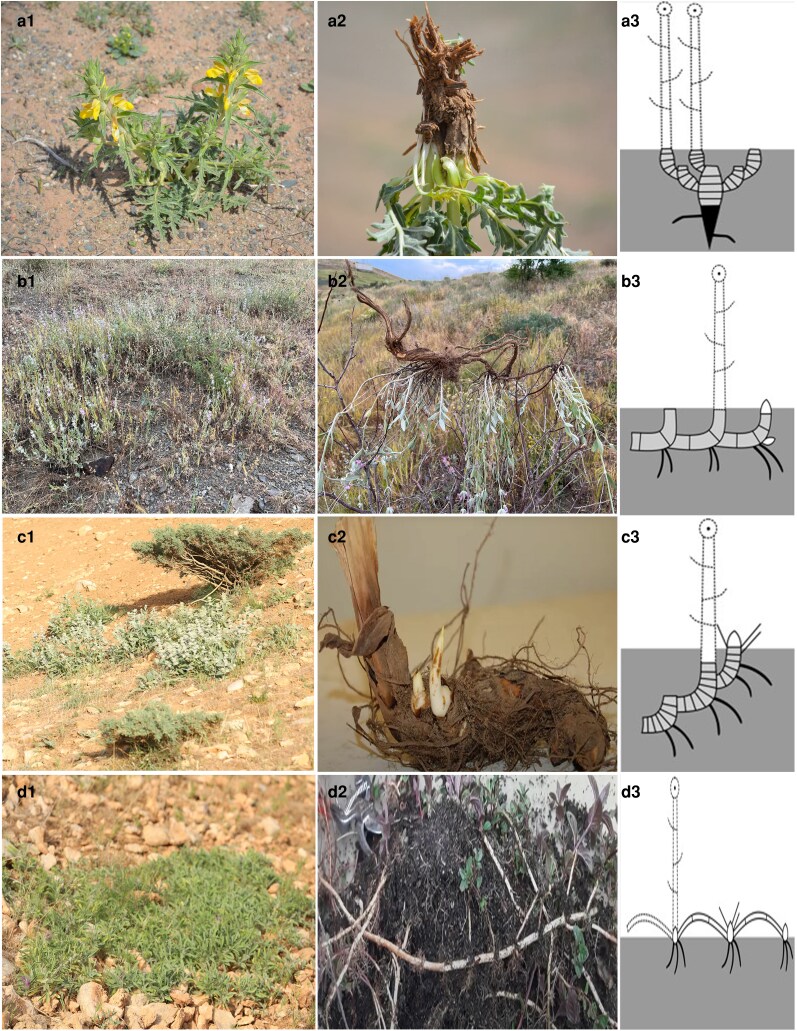
Representative Lamiaceae species illustrating the four BGFs. Photographs show habit (left column), excavated belowground organs (middle column), and schematic diagrams (right column). (a) *Phlomoides laciniata—*main root; (b) *Stachys inflata*—hypogeogenous rhizome; (c) *Phlomis olivieri—*epigeogenous rhizome; (d) *Stachys lavandulifolia—*stoloniferous form.

We assembled data on BGFs for species from Lamiaceae family occurring in the study region. First, we conducted field sampling across various vegetation zones in Iran during the peak vegetation season (March to July) in 2023 and 2024. We sampled 3–5 mature individuals per species from representative sites. Specimens were excavated, cleaned, and preserved in herbaria at Bu-Ali Sina University (BASU). Using dried specimens, we assessed each plant for BGFs according Klimešová et al. (2017). We distinguished three main types of CGOs: epigeogenous rhizomes (formed aboveground, later pulled belowground), hypogeogenous rhizomes (formed directly belowground), and stolons (aboveground stems that root upon contact with the soil). Species lacking these organs, either annual or perennial, were classified as non-clonal plants (Fig. 1).

Additional data on BGFs were supplemented with information from physical herbaria and digital databases, namely the JACQ virtual herbarium (https://www.jacq.org/), the Herbarium of Kurdistan Agricultural and Natural Resources Research and Education Center (HKS), and CLO-PLA3 (Klimešová and Klimeš 2006). From these resources, we included only specimens with detailed records of belowground organs and those occurring in the study region to identify and classify the type of BGFs, ensuring their relevance to our dataset. In total, we assembled data on 281 species from 31 Lamiaceae genera, comprising both clonal and non-clonal species (including annual and perennial herbs).

### Distribution data

The distribution data of the studied species were primarily sourced from the Global Biodiversity Information Facility (GBIF) (https://www.gbif.org) (See Supplementary Table S1). For Iran, additional data were obtained from endemic plant database of Iran (Noroozi et al. 2019b) which mostly are taken from Flora Iranica (Rechinger 1982), then Flora of Iran (Jamzad 2012), and subsequent monographs (see Noroozi et al. 2019a). To ensure data quality, records were systematically cleaned to remove duplicates and inaccuracies following the methods of Maldonado et al. (2015). After cleaning, a total of 29 310 GBIF records were retained to validate the distribution of Lamiaceae species across the region. Individual species had between 4 and 590 records; however, species with fewer than 12 records were excluded, resulting in a final dataset with an average of 174 records per species, sufficient for environmental niche analysis (Drake et al. 2006).

**Figure 2:**
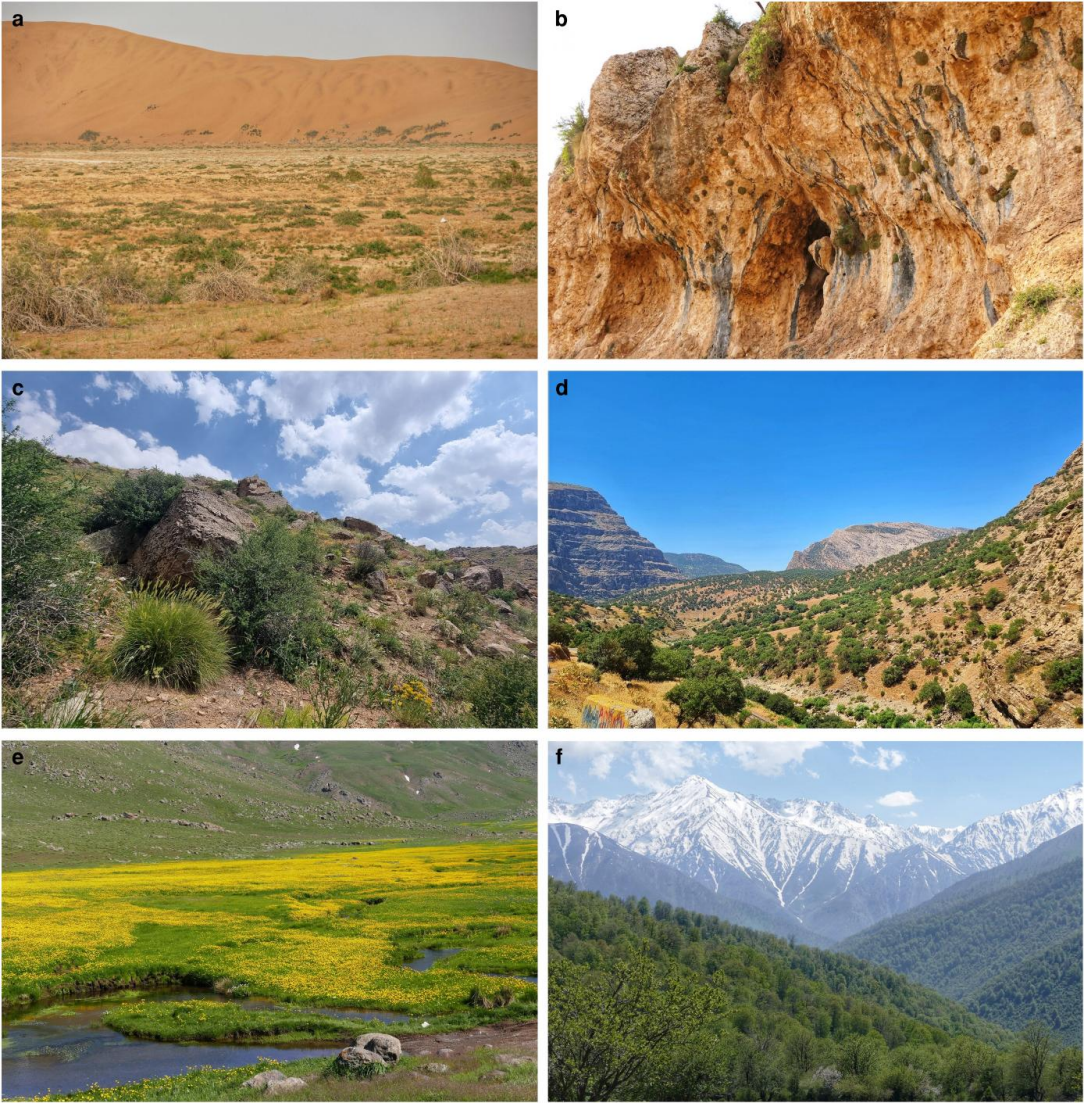
Habitat types of the study: (a) desert and semi-desert steppes (Maranjab Desert, 1000 m a.s.l.), (b) chasmophytic vegetation (Shahu Mts., Northern Zagros, 2500 m a.s.l.), (c) montane steppe shrublands (Cherin Pass, between Sanandaj and Hamadan, 2000 m a.s.l.), (d) woodlands (between Dorud and Bishe waterfall, Central Zagros, 2000 m a.s.l.), (e) wetlands (Talish mountains, 2500 m a.s.l.), and (f) forests (Masichal, Kelardasht, Hyrcanian Forests, Northern Alborz, 2000–2500 m a.s.l.). Photos by. J.N.

In addition to the geographic information, species were classified according to specific habitats within the study area (Fig. 2). For the selection of sites and plant species we used available literature such as all published volumes of ‘Flora Iranica’ edited by Rechinger (1982) and Flora of Iran (Jamzad 2012) and local knowledge of experts and botanists. They were classified into the following habitats: (i) deserts and semi-desert steppes, characterized by extremely low precipitation supporting xerophytic vegetation; (ii) chasmophytic vegetation, found in rocky, mountainous areas where plants thrive in crevices and rocky outcrops; (iii) montane steppe shrublands, occurring at higher elevations with shrubs and herbaceous plants adapted to cooler and often drier conditions; (iv) woodlands, dominated by various species of juniper in areas with moderate to low rainfall, serving as transitional zones between deserts and more humid areas; (v) forests characterized by high humidity and precipitation, supporting broadleaf and mixed forest species; and (vi) wetlands, including marshes and swamps with unique habitats due to water saturation (Noroozi 2020, Table 1).

**Table 1:** Habitats and their environmental niche quantification according to Noroozi (2020).

Habitat	Key characteristics, dominant species	Associated environmental variables	Annual summ of precipitation (mm/year)
Desert and semi-desert steppes	Extremely low precipitation, xerophytic vegetation	Arid soils, high temperature variability	<250
Chasmophytic vegetation	Rocky, mountainous areas; plants in crevices	Rocky terrain, variable moisture	250–500
Montane steppe shrublands	High elevation, shrubs and herbaceous plants	Cool temperatures, well-drained soils	300–600
Woodlands	Juniper-dominated, transitional zones	Moderate to low rainfall, semi-arid soils	400–800
Wetlands	Marshes and swamps, water-saturated habitats	High soil moisture, low drainage	>1000
Forests	High humidity, broadleaf and mixed species	Humid conditions, fertile soils	>800

### Data analysis

We analysed species–environment relationships using climate data at a 5.0-arc-minute resolution (approximately 10 km at the equator) from the CHELSA climatology dataset (version 2.1, 1981–2010) (See Supplementary Table S1) (Karger et al. 2017). After preliminary analysis of all 19 bioclimatic variables, we retained eight precipitation-related variables (BIO12–BIO19), as temperature variables did not significantly influence differences between clonal and nonclonal species. This could be due to the similar temperatures but highly variable precipitation in the different habitats within the study area. The major difference between the habitats in this study is the amount of precipitation and not temperature. For example, the annual precipitation in the Hyrcanian forests, woodlands, shrublands, and desert-semideserts gradually decreases, which is the main reason for the establishment of these macrohabitats. These variables were: BIO12 (annual precipitation), BIO13 (precipitation of the wettest month), BIO14 (precipitation of the driest month), BIO15 (precipitation seasonality), BIO16 (precipitation of the wettest quarter), BIO17 (precipitation of the driest quarter), BIO18 (precipitation of the warmest quarter), and BIO19 (precipitation of the coldest quarter). Because these variables are highly intercorrelated, we summarized them by principal component analysis (PCA).

To summarize the climatic gradients and examine their relationship with species distributions, we performed PCA on the eight precipitation-related variables (BIO12–BIO19) at each geolocated accession site. To determine the effect of these climatic gradients on species distributions, we fitted generalized additive models (GAMs). These models make possible flexible nonlinear response of species occurrence in response to both total precipitation and seasonal variability axes. We fitted these models using binomial distribution. To account for the nonhierarchical nature of the data (species, each of them with potentially many accessions), we fitted the gam models both at the accessions level and at the level of species means, calculated as centroids of values of all accessions of the given species. All analyses were performed using the R package VEGAN (v.2.4-1) (Oksanen et al. 2013) and mgcv (Wood 2017) for GAMs (see Supplementary Methods S1).

Additionally, for phylogenetically controlled analysis, we used Smith and Brown’s (2018) skeleton phylogeny of Angiosperms (the GBOTB.extended phylogeny) (See Supplementary Figure S1), which covers 83 species from our dataset; the remaining species were added by taxonomic information using the *phylo.maker* function (Jin and Qian 2019) with the ‘S3’ scenario. We did not include species from the genera *Lagochilus*, *Pseudodictamnus*, *Moluccella*, and *Hypogomphia* as no information on their position within the family was available. The tree was then used to examine phylogenetic signal in the species mean scores (calculated over all accessions of a given species) along the first and second PCA axes, using the function *phylosig* from the package phytools (v.0.7-90; Revell 2012). Phylogenetic signal in clonality was assessed by fitting a continuous-time Markov model of trait evolution with the function *fitDiscrete* from the package geiger (v.2.0.7; Harmon et al.), and model comparison was conducted with the AIC criterion to evaluate whether equal or different transition rates were more likely.

## Results

A total of 281 species from 31 genera within the family Lamiaceae were analysed. Among these, 166 species (59%), were identified as non-clonal (including both annual and perennial herbs), 73 species (25%) exhibited hypogeogenous rhizomes, 33 species (12%) with epigeogenous rhizomes, and 8 species (3%) having stolon. This diversity in BGFs was distributed across species from various genera within the family.

**Figure 3:**
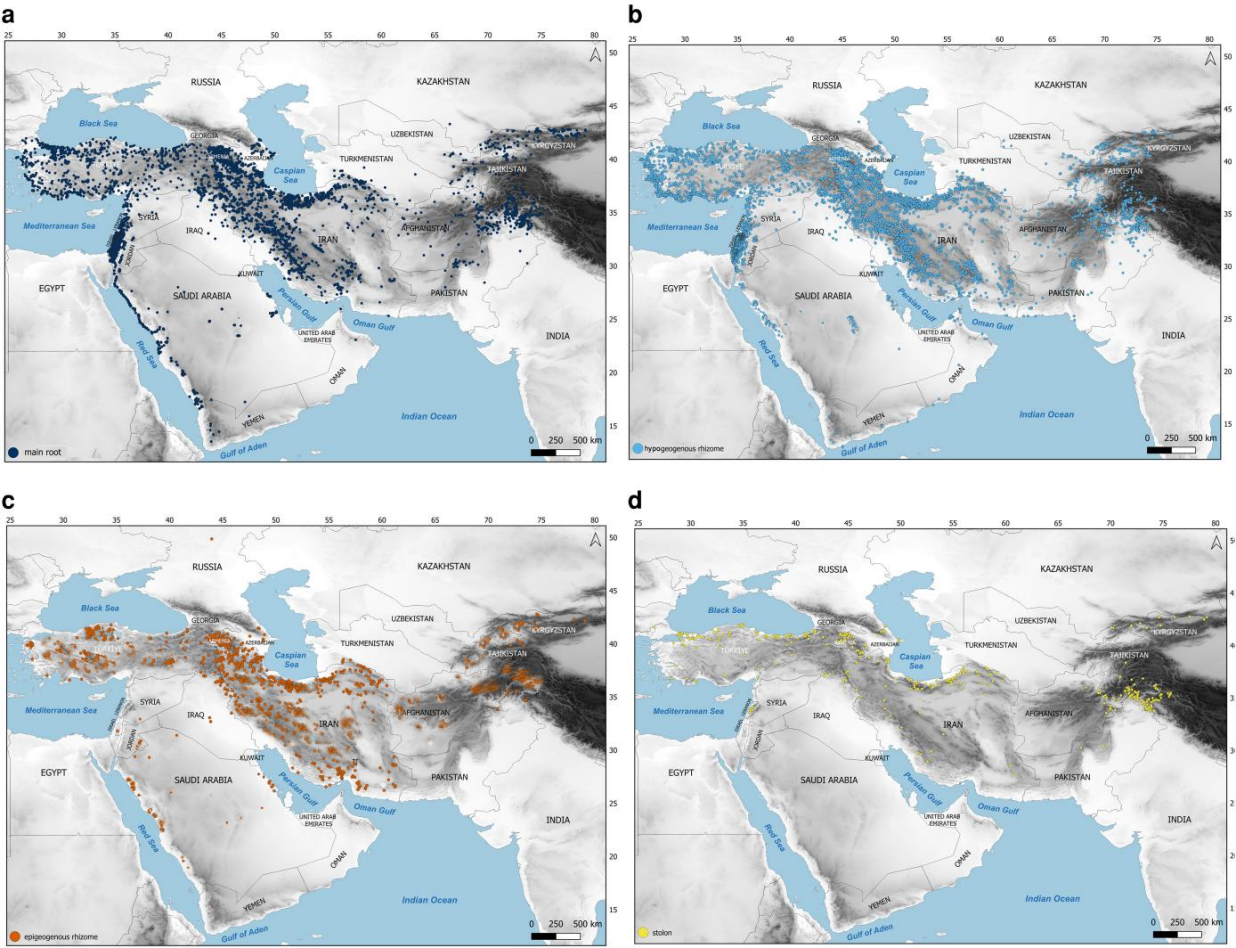
Distribution map of belowground growth forms across SW and Central Asia based on individual Lamiaceae accessions. Belowground growth forms are indicated as: (a) non-clonal species, (b) hypogeogenous rhizomes, (c) epigeogenous rhizomes, and (d) stoloniferous species.

BGFs have different distribution in studied areas (Fig. 3). For example, in arid habitats of southern and central Iran and in Saudi Arabia, hypogeogenous and epigeogenous rhizomes are frequent while, stoloniferous species are overall relatively rare and appear concentrated in localized patches, particularly along river systems and irrigated regions. Non-clonal species exhibit a more widespread distribution across all habitats in SW and Central Asia, suggesting their ability to inhabit diverse environmental conditions, including arid and semi-arid regions, but they are concentrated in the centre of the gradient, where water availability is moderate and variable.

**Figure 4:**
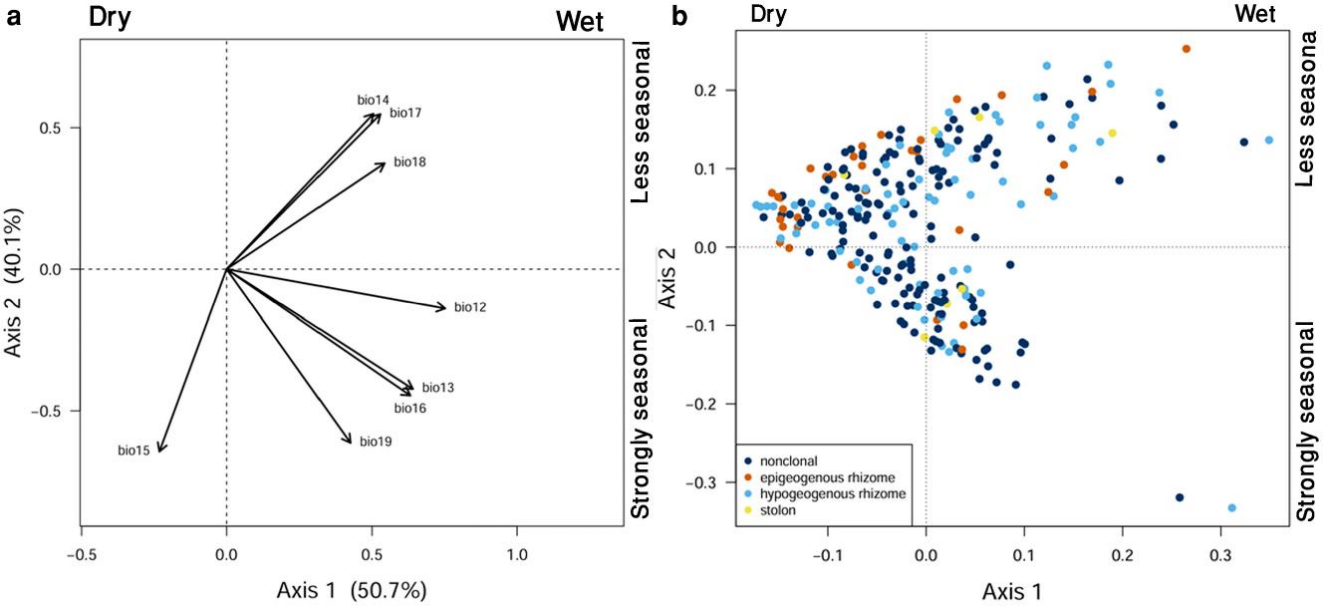
PCA of Lamiaceae accessions based on precipitation-related bioclimatic variables. (a) scores of eight bioclimatic variables (BIO12–BIO19) used for the PCA. BIO12, annual precipitation; BIO13, precipitation of the wettest month; BIO14, precipitation of the driest month; BIO15, precipitation seasonality; BIO16, precipitation of the wettest quarter; BIO17, precipitTation of the driest quarter; BIO18, precipitation of the warmest quarter; and BIO19, precipitation of the coldest quarter. (b) positions of species mean in the space of these axes with individual BGF indicated by colours: dark blue (non-clonal species), sky blue (hypogeogenous rhizomes), orange (epigeogenous rhizomes), and yellow (stoloniferous species).

To summarize climatic gradients and examine their relationship with species distributions, we performed a PCA on the eight precipitation-related variables (BIO12–BIO19) at each geolocated accession site (Fig. 4). The first two axes accounted for 90.7% of the total variance and were retained for further analyses. Axis 1 (50.7% of variance) represented overall precipitation, with strong positive loadings from annual precipitation (BIO12), precipitation of the wettest month (BIO13), wettest quarter (BIO16), and coldest quarter (BIO19), describing a gradient from dry to wet climates from left to right (Fig. 4a). Axis 2 (40.0% of variance) captured seasonal precipitation patterns, contrasting precipitation of the driest month (BIO14), driest quarter (BIO17), and warmest quarter (BIO18) with precipitation seasonality (BIO15). This axis therefore represents a gradient from more seasonal to less seasonal precipitation regimes from bottom to top, reflecting species’ responses to varying wet and dry periods. The positions of species mean in the space of these axes illustrate the distribution of individual BGFs, with each growth form indicated separately (Fig. 4b).

**Figure 5:**
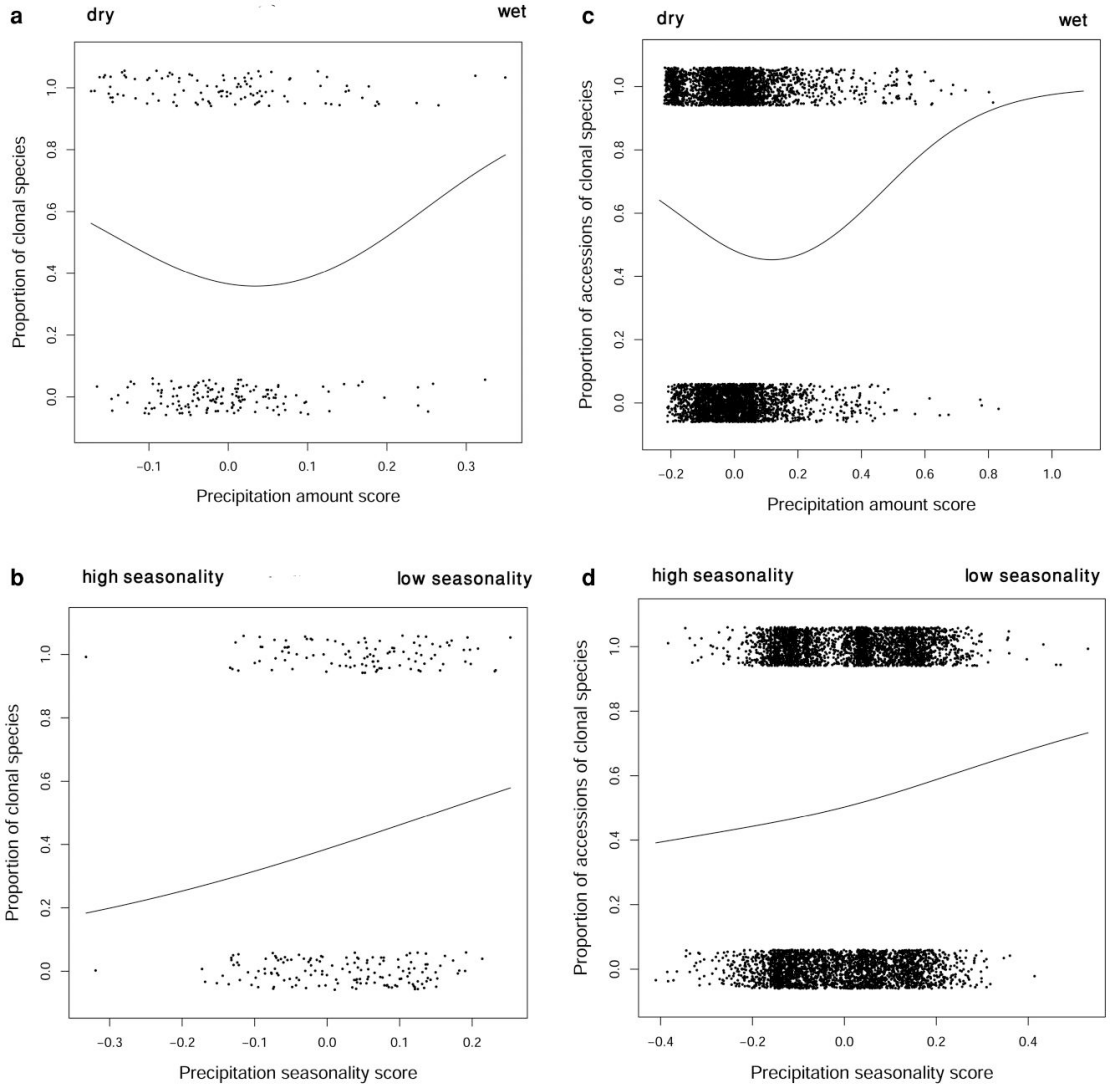
GAM analysis. Analyses on species means (a and b) and individual accessions (c and d). (a and c) GAM fits on the first principal component axis scores, which express the overall precipitation sums. (b and d) GAM fits on the second principal component axis scores, which express precipitation seasonality. Black points in (a) and (b) represent individual species, while in (c) and (d), they represent individual accessions (randomly sampled 20% of accessions shown).

The relationship between overall precipitation captured by the first two PCA axes score and the proportion of clonality analysed by GAM showed a clear nonlinear relationship, suggesting that clonality initially decreases with increasing precipitation but subsequently increases as precipitation continues to rise (Fig. 5). Specifically, at lower precipitation ranges (negative PCA scores), clonality is relatively high but declines to its lowest point around moderate precipitation values (PCA score ≈ 0). Beyond this threshold, clonality increases steadily, reaching its maximum at the highest precipitation levels (positive PCA scores). The same pattern is shown by the analyses on species means (Fig. 5a and b) and on individual accessions (Fig. 5c and d). The second PCA axis score (Fig. 5b and d) captures precipitation seasonality and shows significant monotonous relationship to the proportion of clonal species. As precipitation seasonality decreases, the proportion of clonality increases. The statistical support for these patterns is summarized in Table 2, which presents the GAM test statistics, associated *P*-values, and adjusted *R*² values corresponding to the relationships shown in Fig. 5.

**Table 2:** Results of the GAMs presented in Fig. 4 based on species means and individual accessions: PCA axis 1 (precipitation amount) and PCA axis 2 (precipitation seasonality).

Analysis type	Axis	Chi sq	*P* value	*R*² (adj)
Species based	1	5.848	0.0526	0.0196
Species based	2	5.641	0.0176	0.0178
Accessions based	1	337.2	<2e−16	0.0129
Accessions based	2	251.6	<2e−16	0.00904

**Figure 6:**
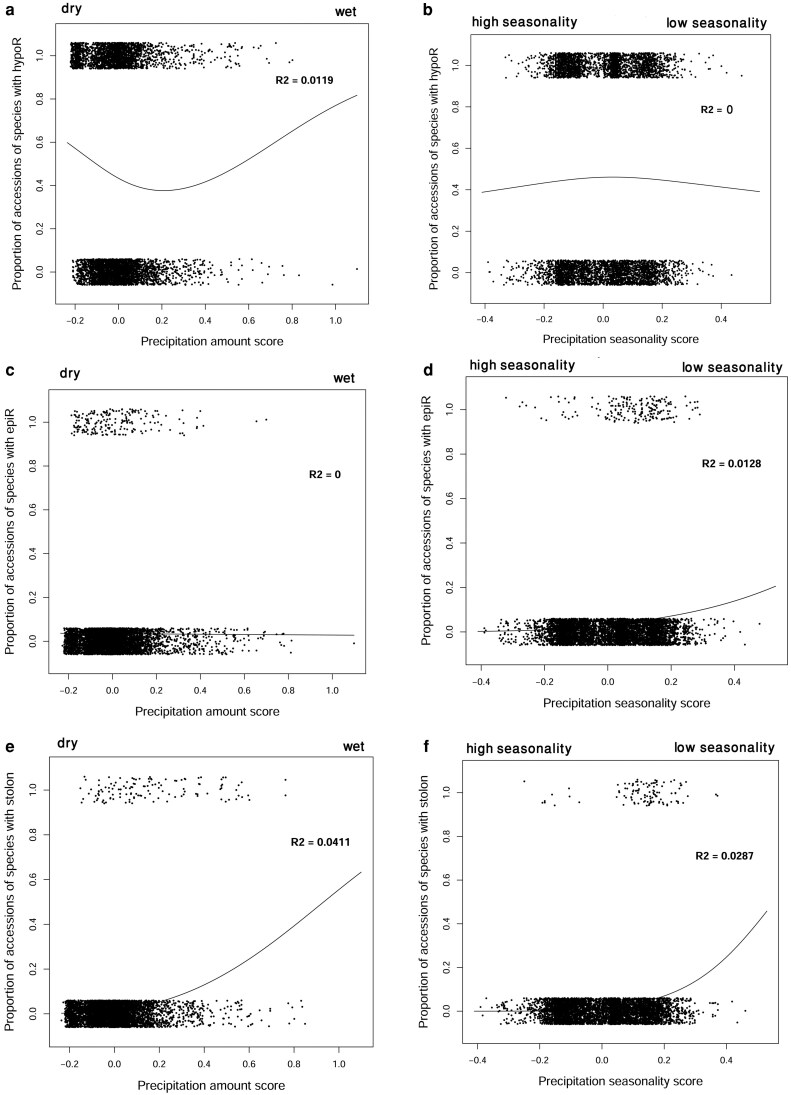
GAM fits showing the relationships between CGOs and the first two principal components of precipitation: PC1 (overall precipitation amount) and PCA2 (precipitation seasonality). (a and b) Hypogeogenous rhizomes, (c and d) epigeogenous rhizomes, and (e and f) stolons. Black points indicate individual species accessions (randomly sampled 20% of accessions shown).

**Figure 7:**
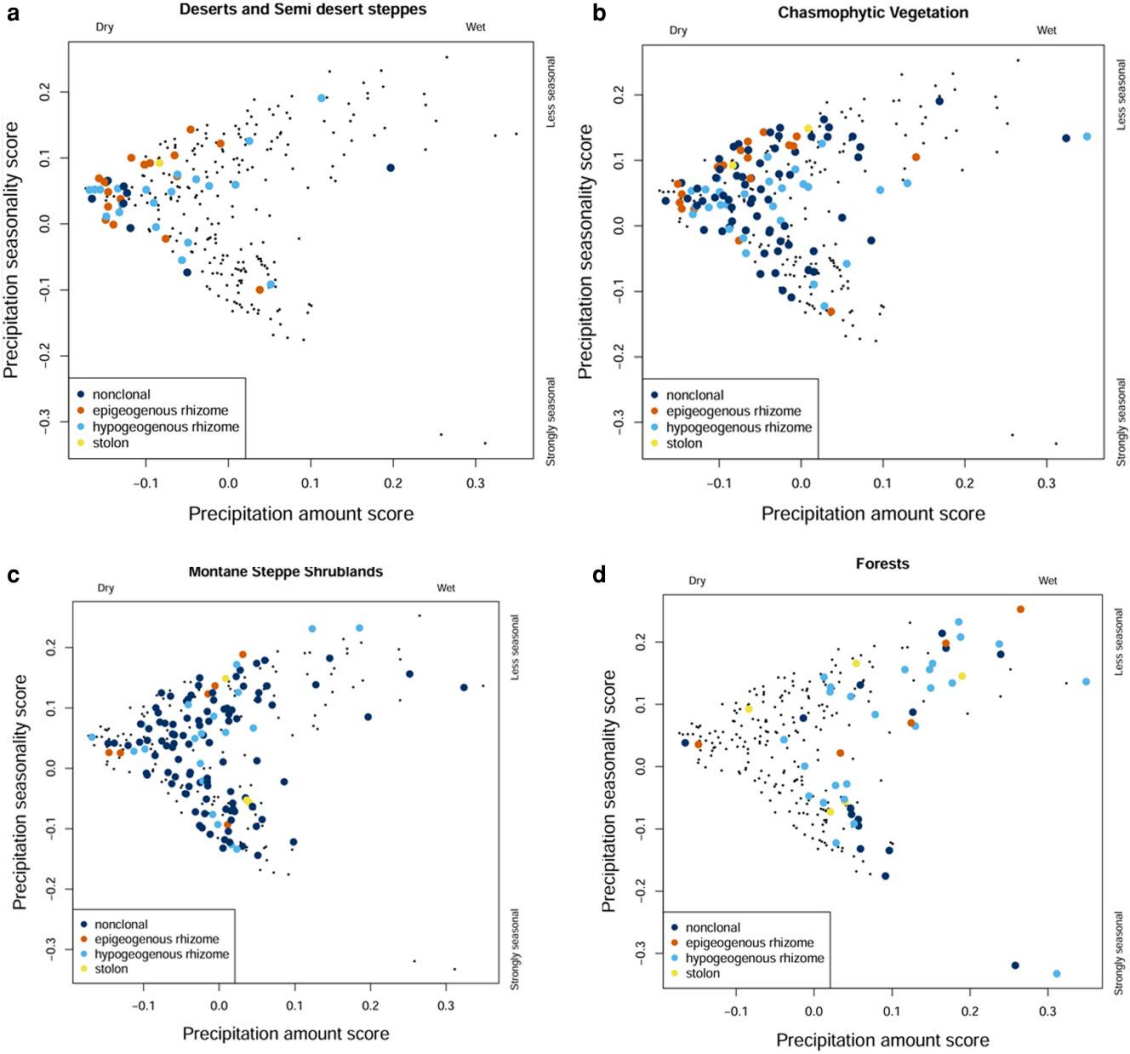
PCA of Lamiaceae species distribution by BGFs in the space of precipitation variables (see Fig. 4 for the relationship between the axes and the BIO variables). Each point represents a species (centroid of PCA positions of all accessions of that species), with highlighted points indicating species occurring in the given habitat. (a) Desert steppes, (b) chasmophytic vegetation, (c) montane steppe shrublands, and (d) forests.

**Figure 8:**
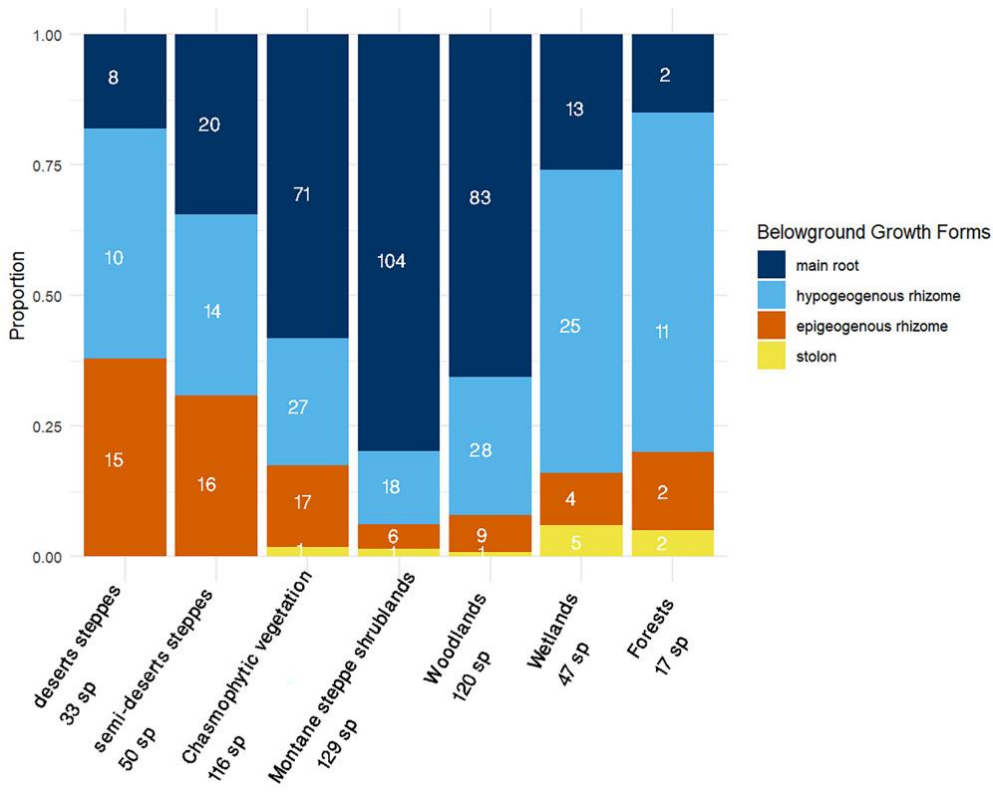
Proportional representation of BGFs of Lamiaceae species across different habitat types in southwest and Central Asia. Colours indicate four BGFs: main root (dark blue), hypogeogenous rhizome (light blue), epigeogenous rhizome (orange), and stolon (yellow). Numbers inside bars show the count of species with each BGF within a given habitat. Total species numbers per habitat are indicated below the *x*-axis labels.

To complement the combined analyses, we also examined each CGO separately. The additional GAM plots (Fig. 6) show that hypogeogenous rhizomes display little association with precipitation, epigeogenous rhizomes respond weakly to precipitation seasonality, and stolons respond to both precipitation amount and seasonality. Consequently, the distribution of clonal and non-clonal species varies across habitats (Figs. 7 and 8). In arid habitats, clonal species are more prevalent. In desert steppes with 33 species in total, clonal species—including those with hypogeogenous rhizomes, epigeogenous rhizomes, and stolons—comprise 25 species (76%), leaving only 8 species (24%) as non-clonal. A similar pattern is observed in semi-desert steppes (50 species), where clonal species account for 30 species (63%) compared to 20 species (37%) that are non-clonal.

In chasmophytic vegetation (116 species), non-clonal species dominate, comprising 71 species (61%), while clonal species account for 45 species (39%). A similar pattern is observed in montane steppes shrublands (129 species), where non-clonal species comprise 104 species (81%) and clonal species—including those with hypogeogenous rhizomes, epigeogenous rhizomes, and stolons—account for 25 species (19%). In woodlands (120 species), non-clonal species represent 83 species (69%), with clonal species accounting for 37 species (31%).

Forests (47 species) and wetlands (17 species) also show a strong dominance of clonal species. In forests, clonal species make up 43 species (91%), with just 4 species (9%) being non-clonal. Wetlands exhibit a similar trend, with clonal species comprising 15 species (88%), while non-clonal species contribute only 2 species (12%).

Among clonal species, hypogeogenous rhizomes are particularly prevalent in Forests, where they contribute 25 species (53%), wetlands (11 species, 65%), semi-desert steppes (17 species), and woodlands (28 species). This suggests a significant presence of hypogeogenous rhizomes in habitats with higher moisture availability or transitional habitats.

Both epigeogenous and hypogeogenous rhizomes are common in desert steppes and semi-desert steppes. In desert steppes, epigeogenous rhizomes account for 15 species (45%), while hypogeogenous rhizomes contribute 10 species (30%). Similarly, in semi-desert steppes, epigeogenous rhizomes represent 16 species (29%) and hypogeogenous rhizomes account for 14 species (28%).

In addition, we assessed whether these patterns showed evidence of phylogenetic signal. There was no phylogenetic signal in species means along the first (precipitation amount; lambda = 0.0) and second (precipitation seasonality; lambda = 0.02) axes. Neither lambda value was significantly different from zero. There was a weak phylogenetic signal in clonality (lambda = 0.431, assuming the equal rates transition process).

## Discussion

Our study reveals a pattern in clonal reproduction strategies across environmental gradients in SW and Central Asia. Analyses of BGFs, species distribution, and precipitation-related bioclimatic variables indicate that clonality is prevalent at both ends of the precipitation/wetness gradient, i.e. both in dry and wet ecosystems, and increase towards lower precipitation seasonality. The pattern was the same for precipitation gradient as well as for habitat conditions. These results show that clonal species from Lamiaceae family may successfully cope with drought stress and that indicate that the occurrence of clonal species, and especially hypogeogenous rhizomes, is not restricted to moist places. We therefore rejected our hypothesis based on results from Central Europe.

### BGF diversity in Lamiaceae family

The CGOs found in representatives from Lamiaceae family growing in SW and Central Asia were rhizomes, consistent with those found in Central Europe (Klimešová and Klimeš 2008). The ratio of epigeogenous to hypogeogenous rhizomes was similar across these regions, despite the potentially more suitable conditions for hypogeogenous rhizomes in waterlogged wetland soils compared to terrestrial habitats (Philbrick and Les 1996, Klimešová et al. 2012). The third CGO type found in studied family were stolons that are also common in Central Europe. The proportion of plants with stolons was 2% in the study region, closely matching the 5% observed in Central Europe (Sosnová et al. 2010). Contrary to Central Europe other common modes of clonal growth were not found. For example, root sprouting that occurs also in species from Lamiaceae from Europe (*Ajuga genevensis*) was not recorded in studied region.

### Clonality at the arid end of the gradient

Our results showed that clonal plants increase in representation under conditions of lower water availability. This pattern was consistent not only along the broad precipitation gradient in the Middle East but also across local habitat contrasts, where clonal species were more frequent in drier habitat types. This is a novel finding, as earlier research on clonality along wetness gradients has shown clonal species to dominate wet habitats, both along local gradient within one macroclimate (Klimeš et al. 1997, Klimešová et al. 2012, Klimešová et al. 2011, Song and Ming 2002) or spanning broad climatic gradients (Ye et al. 2014). It should be noted that studies spanning broad climatic gradients prohibit deeper understanding of clonal growth strategies as they do not provide information about occurrence of clonal species in local habitats (differing, e.g. in water availability).

By integrating both habitat-level and broad-gradient perspectives, our study extends these findings by demonstrating that in the Middle East, clonality responds strongly to water availability across multiple scales, and that the response is strongly nonlinear, with two maxima at both ends of the aridity gradient. In arid regions, water and nutrient scarcity present significant challenges to plant survival and reproduction and clonality may provide critical adaptive advantages, enabling both resource storage and vegetative reproduction. Although mechanisms of this should be experimentally tested, we could speculate that hypogeogenous rhizomes, a prevalent CGO in such environments, enhance plant resilience by storing essential resources, including water, within below-ground structures, thus supporting survival during extended drought periods (Sánchez-Gómez et al. 2006, Klimešová et al 2023, Qi and Zhao 2024). Moreover, these rhizomes may facilitate the exploration of greater soil volumes by extending through the substrate and producing new ramets at distant locations, allowing plants to access dispersed water and nutrient resources. Likewise, epigeogenous rhizomes can possibly play a key role in drought resistance by facilitating rapid regrowth following disturbances, thereby ensuring the persistence of clonal species under harsh environmental conditions (Wang et al. 2018). In contrast, stolons cannot serve any of these functions and therefore are missing at the arid end of the gradient.

On the population level, nonclonal species rely entirely on seed reproduction, it typically suffers from scarcity of favourable years under which successful seedling reproduction can take place (Kitchen et al. 2015, Bogdziewicz 2021). Clonal offspring, on the other hand, are much better equipped to survive such periods and, thus, often outperform sexually produced offspring, establishing more quickly, which is advantageous under time-constrained growing seasons (Mandujano et al. 2010). By mitigating the risks associated with sexual reproduction, clonality may provide an additional important buffer against environmental variability, ensuring the survival and resilience of species in arid habitats.

### Clonality at the wet end of the gradient

Evidence of this study suggests that clonality can also thrive in resource-abundant ecosystems like wetlands and forests. This confirms earlier findings (Klimešová et al. 2011, Klimešová and Herben 2015, Klimešová et al. 2018, Sosnová et al. 2010, Ye et al. 2020). In these communities, plants must cope with several stress factors, primarily high productivity resulting in competition for light and difficulties with sexual reproduction. Clonal plants can dominate competitive settings by spreading extensively clonally, maintaining stable populations, and effectively occupying available niches (Grace 1993, Klimešová et al. 2018, Doležal et al. 2020). The similarities in representation of individual CGOs between SW and Central Asia and Central Europe (Klimešová and Klimeš 2008, Sosnová et al. 2010) suggest that these strategies are not unique to arid regions but represent a broader ecological pattern. The ability of clonal plants to dominate wetlands and forests highlights their versatility and ecological significance in maintaining population stability under environmental conditions associated with competition for light.

It is important to note that different CGOs have specific roles in ecosystem functioning. They influence key processes, such as nutrient cycling, soil stabilization, and vegetation dynamics, especially in regions where water availability is a limiting factor. Clonal traits can enhance ecosystem resilience by improving vegetation cover, reducing erosion, and maintaining stability under stress (Klimešová et al. 2023) and by homogenizing resource availability (Magyar et al. 2007). Furthermore, variation in clonal strategies can promote biodiversity through spatial niche differentiation and resource partitioning (Richardson and Pyšeket al. 2012).

### Limitations of the study

It should be noted that the findings here concern only one, albeit species-rich family and concern only one region with a long water availability gradient, although one of the major ones. From previous studies we know that pattern of clonality for individual taxonomic groups may differ. In Japan, species-specific analyses showed that clonality is constrained not only by climate but also by phylogenetic history, with different lineages showing distinct propensities for clonal growth (Fujinuma et al. 2019). Similarly, study by Ye et al. (2016) revealed distinct patterns of clonality proportion in relation to environmental gradients for representatives of different taxonomic groups across China. Our exclusive focus on the Lamiaceae family, though advantageous due to its diversity and clonal versatility, should be considered when extrapolation of these findings is done to the whole Angiosperm lineage. Expanding future research to include additional families and biogeographic regions would enhance our understanding of the diversity and distribution of clonal strategies across plant lineages and ecosystems.

## Conclusions

This study underscores the occurrence of clonality across contrasting environments, from arid deserts to resource-abundant wetlands and suggests that clonality may not be so rare in arid environments as previously thought and challenge our understanding how clonality responds to water scarcity. Future research should therefore focus on identifying mechanisms through which clonal growth contributes to plant resilience under varying precipitation patterns. For instance, detailed investigations into how clonal traits like rhizome extension or resource storage capacity interact with changing seasonal rainfall and drought events could provide actionable insights. Regional studies in data-scarce areas, particularly in biodiversity hotspots or regions highly sensitive to climate variability, are crucial. Additionally, experimental studies that manipulate water availability in controlled environments or long-term field observations would help elucidate how shifts in precipitation regimes influence the balance between sexual and clonal reproduction. By advancing knowledge of these processes, this research contributes to more targeted strategies for conserving biodiversity and managing ecosystems in the face of global environmental change. Specifically, understanding clonal traits can help prioritize species with higher resilience in restoration projects, guide the selection of planting materials in degraded habitats, and improve predictive models of vegetation dynamics under changing precipitation regimes.

## Supplementary Material

plaf069_Supplementary_Data

## Data Availability

The raw data and R codes are available as supporting information.
