# Supplementary material for: Plants clonal strategies are well associated with aridity gradients: insights from Lamiaceae family in the SW and Central Asia
Source: AoB Plants. 2025 Dec 8;17(6):plaf069. doi: 10.1093/aobpla/plaf069 (PMC12723225; doi:10.1093/aobpla/plaf069)
Supplement: plaf069_Supplementary_Data [file plaf069_supplementary_data.zip › Methods S1.pdf]

Methods S1. R workflow used for data analysis

This document provides the R code used to reproduce the principal component analysis (PCA), generalized additive models (GAMs) and bar plot (habitat representation), used in the study.

PCA and GAM analysis

```
library(mgcv)

library(vegan)

library(lme4)


source("plot.pca.R")


hypocolor   = "#56B4E9"

epicolor    = "#D55E00"

noncloncolor  = "#003366"

stoloncolor  = "#F0E442"


plette = c(noncloncolor, epicolor, hypocolor, stoloncolor)


dta <- read.table("accession-based analysis.txt", sep="\t",header=T)


# Inspect the structure of the data


# Reclassify Dominant_CGO: "main root" -> non_clonal (0), others -> clonal (1)
```

```
dta$clonal <- ifelse(dta$Dominant_CGO %in% c("main root","non-clonal"), 0, 1)

dta$hypoR <- ifelse(dta$Dominant_CGO == "hypogeogenous rhizome", 1,0)

dta$epiR <- ifelse(dta$Dominant_CGO == "epigeogenous rhizome", 1,0)

dta$stolon <- ifelse(dta$Dominant_CGO == "stolon", 1,0)


#Histograms


#select bio variables

bio_ind = grep("bio",colnames(dta),fixed=T)

bio_vars = dta[,bio_ind]

bio_prec = bio_vars[,4:11] #subset precipitation variables


bio_precsc = scale(bio_prec)  #scale bio vars


rdout_prec = rda(bio_precsc)  #run the pca


plot(rdout_prec,display="sp") #show the plot of precvars


eigenvals(rdout_prec)/sum(eigenvals(rdout_prec))  #show the proportion of variation explained y individual axes


dta$scrprec1 = scores(rdout_prec)$si[,1]  #extract positions of individual accessions on the first two axes

dta$scrprec2 = scores(rdout_prec)$si[,2]


dta$species = sapply(dta$Species,gsub,pattern=" ",replacement="_",fixed=T)  #generate new species names, note the lowercase s in the left-hand side
```

```
#find species with frequency over 10

limitfreq = 10 #could be changed if needed
specfreq = table(dta$species)  #find species frequencies
uze = dta$species %in% names(specfreq)[specfreq >= limitfreq]
dtasubset = dta[uze,]  #subset of data with species with frequency > limitfreq

dta$speciesF = as.factor(dta$species)  #species names as factors if needed
dtasubset$speciesF = as.factor(dtasubset$species)

#calculate means for means-based analysis
mnsprec1 = tapply(dta$scrprec1,dta$species,mean)
mnsprec2 = tapply(dta$scrprec2,dta$species,mean)
mnsclon = tapply(dta$clon,dta$species,mean)
mnsepiR = tapply(dta$epiR,dta$species,mean)
mnshypoR = tapply(dta$hypoR,dta$species,mean)
mnsstolon = tapply(dta$stolon,dta$species,mean)
mnuze = names(mnsprec1) %in% names(specfreq)[specfreq >= limitfreq]
mnsCGO = mnsepiR + 2 * mnshypoR + 3 * mnsstolon

#aggregate habitats for species
habind = grepl(".",colnames(dta),fixed=T) | colnames(dta) %in% c("Wetlands","Forests" )
habitats = aggregate(dta[,habind],list(dta$species),mean)
rownames(habitats) = habitats[,1]
habitats = habitats[,-1]
```

```
habitats[is.na(habitats)] = 0

habitats[habitats == 0.5] = 1


#calculate species cetroids in the PCA space for species-based analysis

mnscore1 = tapply(dta$scrprec1,dta$species,mean)

mnscore2 = tapply(dta$scrprec2,dta$species,mean)


#PCA plot


pdf("PCA-1.pdf")


pos = ifelse(scores(rdout_prec)$species[,1] > 0, 4,2)

pos["bio16"] = 1 #put the label below

pos["bio14"] = 3 #put the label above

plot.pca(rdout_prec,pts=F,xpd=T,pos.sp=pos,fctx=0.1,fcty=0.1,las=1)


tke = runif(nrow(dta)) < 0.1

plot.pca(rdout_prec,pts=F,xpd=T,pos.sp=pos,fctx=0.1,fcty=0.1,las=1)

points(dta$scrprec1[tke],dta$scrprec2[tke],cex=0.3,pch=3)


plot(mnscore1,mnscore2,col = plette[mnsCGO+1],xlab="Axis 1",ylab="Axis
2",las=1,pch=19,cex.lab=1.4 )

abline(v=0,lty=3)

abline(h=0,lty=3)

legend("bottomleft", col=plette,pch=19,legend=c("nonclonal","epigeogenous
rhizome","hypogeogenous rhizome", "stolon"),cex=0.85)
```

```
dev.off()



#end of initial calculations

#*************************


#nonhierarchical fits with accessions


pdf("Nonhierarchical.pdf")


gam_model <- gam(clonal ~ s(scrprec1,k=3),
                 family = binomial,
                 data = dta)


prec_range <- seq(min(dta$scrprec1, na.rm = TRUE), max(dta$scrprec1, na.rm = TRUE), length.out = 100)
new_dta <- data.frame(prec_range)
colnames(new_dta) <- "scrprec1"  # Ensure the column name matches the model variable
preds <- predict(gam_model, newdata = new_dta, type = "response")


plot(prec_range,preds, type="l",xlab="Precipitation amount score",ylab="Proportion of accessions of clonal species",cex.lab=1.3)


summary(gam_model)


# with species points
```

```
plot(prec_range,preds, type="l",xlab="Precipitation amount score",ylab="Proportion of accessions of clonal species",cex.lab=1.3,ylim=c(-0.1,1.1))

tke = runif(length(dta$scrprec1)) < 0.2

points(dta$scrprec1[tke],jitter(dta$clonal[tke],factor=0.3),pch=19,cex=0.3)




gam_model <- gam(clonal ~ s(scrprec2,k=3),
                 family = binomial,
                 data = dta)


prec_range <- seq(min(dta$scrprec2, na.rm = TRUE), max(dta$scrprec2, na.rm = TRUE), length.out = 100)

new_dta <- data.frame(prec_range)

colnames(new_dta) <- "scrprec2"  # Ensure the column name matches the model variable

preds <- predict(gam_model, newdata = new_dta, type = "response")


plot(prec_range,preds, type="l",xlab="Precipitation seasonality score",ylab="Proportion of accessions of clonal species",cex.lab=1.3)


summary(gam_model)


# with species points


plot(prec_range,preds, type="l",xlab="Precipitation seasonality score",ylab="Proportion of accessions of clonal species",cex.lab=1.3,ylim=c(-0.1,1.1))

tke = runif(length(dta$scrprec1)) < 0.2
```

```
points(dta$scrprec2[tke],jitter(dta$clonal[tke],factor=0.3),pch=19,cex=0.3)

dev.off()

#nonhierarchical fits of individual CGO with accessions

pdf("Nonhierarchical-CGO.pdf")

gam_model <- gam(hypoR ~ s(scrprec1,k=3),
          family = binomial,
          data = dta)

prec_range <- seq(min(dta$scrprec1, na.rm = TRUE), max(dta$scrprec1, na.rm = TRUE),
length.out = 100)
new_dta <- data.frame(prec_range)
colnames(new_dta) <- "scrprec1"  # Ensure the column name matches the model variable
preds <- predict(gam_model, newdata = new_dta, type = "response")

plot(prec_range,preds, type="l",xlab="Precipitation amount score",ylab="Proportion of
accessions of species with hypoR",cex.lab=1.3)
```

```
summary(gam_model)

# with species points

plot(prec_range,preds, type="l",xlab="Precipitation amount score",ylab="Proportion of accessions of species with hypoR",cex.lab=1.3,ylim=c(-0.1,1.1))

tke = runif(length(dta$scrprec1)) < 0.2

points(dta$scrprec1[tke],jitter(dta$hypoR[tke],factor=0.3),pch=19,cex=0.3)

title(main=paste("R2 =",round(summary(gam_model)$r.sq,4)))



gam_model <- gam(hypoR  ~ s(scrprec2,k=3),

                family = binomial,

                data = dta)


prec_range <- seq(min(dta$scrprec2, na.rm = TRUE), max(dta$scrprec2, na.rm = TRUE), length.out = 100)

new_dta <- data.frame(prec_range)

colnames(new_dta) <- "scrprec2"  # Ensure the column name matches the model variable

preds <- predict(gam_model, newdata = new_dta, type = "response")


plot(prec_range,preds, type="l",xlab="Precipitation seasonality score",ylab="Proportion of accessions of species with hypoR",cex.lab=1.3)


summary(gam_model)
```

```
# with species points

plot(prec_range,preds, type="l",xlab="Precipitation seasonality score",ylab="Proportion of accessions of species with hypoR",cex.lab=1.3,ylim=c(-0.1,1.1))

tke = runif(length(dta$scrprec1)) < 0.2

points(dta$scrprec2[tke],jitter(dta$hypoR[tke],factor=0.3),pch=19,cex=0.3)

title(main=paste("R2 =",round(summary(gam_model)$r.sq,4)))



gam_model <- gam(epiR ~ s(scrprec1,k=3),

                 family = binomial,

                 data = dta)


prec_range <- seq(min(dta$scrprec1, na.rm = TRUE), max(dta$scrprec1, na.rm = TRUE), length.out = 100)

new_dta <- data.frame(prec_range)

colnames(new_dta) <- "scrprec1"  # Ensure the column name matches the model variable

preds <- predict(gam_model, newdata = new_dta, type = "response")


plot(prec_range,preds, type="l",xlab="Precipitation amount score",ylab="Proportion of accessions of species with epiR",cex.lab=1.3)


summary(gam_model)


# with species points
```

```
plot(prec_range,preds, type="l",xlab="Precipitation amount score",ylab="Proportion of accessions of species with epiR",cex.lab=1.3,ylim=c(-0.1,1.1))

tke = runif(length(dta$scrprec1)) < 0.2

points(dta$scrprec1[tke],jitter(dta$epiR[tke],factor=0.3),pch=19,cex=0.3)

title(main=paste("R2 =",round(summary(gam_model)$r.sq,4)))



gam_model <- gam(epiR  ~ s(scrprec2,k=3),

        family = binomial,

        data = dta)


prec_range <- seq(min(dta$scrprec2, na.rm = TRUE), max(dta$scrprec2, na.rm = TRUE), length.out = 100)

new_dta <- data.frame(prec_range)

colnames(new_dta) <- "scrprec2"  # Ensure the column name matches the model variable

preds <- predict(gam_model, newdata = new_dta, type = "response")


plot(prec_range,preds, type="l",xlab="Precipitation seasonality score",ylab="Proportion of accessions of species with epiR",cex.lab=1.3)


summary(gam_model)


# with species points


plot(prec_range,preds, type="l",xlab="Precipitation seasonality score",ylab="Proportion of accessions of species with epiR",cex.lab=1.3,ylim=c(-0.1,1.1))

tke = runif(length(dta$scrprec1)) < 0.2
```

```
points(dta$scrprec2[tke],jitter(dta$epiR[tke],factor=0.3),pch=19,cex=0.3)

title(main=paste("R2 =",round(summary(gam_model)$r.sq,4)))



gam_model <- gam(stolon ~ s(scrprec1,k=3),
                 family = binomial,
                 data = dta)

prec_range <- seq(min(dta$scrprec1, na.rm = TRUE), max(dta$scrprec1, na.rm = TRUE),
length.out = 100)

new_dta <- data.frame(prec_range)

colnames(new_dta) <- "scrprec1"  # Ensure the column name matches the model variable

preds <- predict(gam_model, newdata = new_dta, type = "response")

plot(prec_range,preds, type="l",xlab="Precipitation amount score",ylab="Proportion of
accessions of species with stolon",cex.lab=1.3)

summary(gam_model)

# with species points

plot(prec_range,preds, type="l",xlab="Precipitation amount score",ylab="Proportion of
accessions of species with stolon",cex.lab=1.3,ylim=c(-0.1,1.1))

tke = runif(length(dta$scrprec1)) < 0.2

points(dta$scrprec1[tke],jitter(dta$stolon[tke],factor=0.3),pch=19,cex=0.3)

title(main=paste("R2 =",round(summary(gam_model)$r.sq,4)))
```

```
gam_model <- gam(stolon  ~ s(scrprec2,k=3),
                family = binomial,
                data = dta)

prec_range <- seq(min(dta$scrprec2, na.rm = TRUE), max(dta$scrprec2, na.rm = TRUE),
length.out = 100)

new_dta <- data.frame(prec_range)

colnames(new_dta) <- "scrprec2"  # Ensure the column name matches the model variable

preds <- predict(gam_model, newdata = new_dta, type = "response")

plot(prec_range,preds, type="l",xlab="Precipitation seasonality score",ylab="Proportion of
accessions of species with stolon",cex.lab=1.3)

summary(gam_model)

# with species points

plot(prec_range,preds, type="l",xlab="Precipitation seasonality score",ylab="Proportion of
accessions of species with stolon",cex.lab=1.3,ylim=c(-0.1,1.1))

tke = runif(length(dta$scrprec1)) < 0.2

points(dta$scrprec2[tke],jitter(dta$stolon[tke],factor=0.3),pch=19,cex=0.3)

title(main=paste("R2 =",round(summary(gam_model)$r.sq,4)))

dev.off()
```

```
#predictions with means only, all species

pdf("Means_points.pdf")

gam_model_means <- gam(mnsclon ~ s(mnsprec1,k=3),
          family = binomial)
bio_range <- seq(min(mnsprec1, na.rm = TRUE), max(mnsprec1, na.rm = TRUE), length.out
= 100)
new_dta <- data.frame(bio_range)
colnames(new_dta) <- "mnsprec1"  # Ensure the column name matches the model variable

summary(gam_model_means)

preds_means = predict(gam_model_means, newdata = new_dta, type = "response")

plot(bio_range,preds_means, type="l",xlab="Precipitation amount score",ylab="Proportion
of clonal species",cex.lab=1.3)

# with species points

plot(bio_range,preds_means, type="l",xlab="Precipitation amount score",ylab="Proportion
of clonal species",cex.lab=1.3,ylim=c(-0.1,1.1))
tke = runif(length(mnsprec1)) < 0.9
points(mnsprec1[tke],jitter(mnsclon[tke],factor=0.3),pch=19,cex=0.3)
title(main=paste("R2 =",round(summary(gam_model_means)$r.sq,4)))
```

```
#second component

gam_model_means <- gam(mnsclon ~ s(mnsprec2,k=3),
            family = binomial)
bio_range <- seq(min(mnsprec2, na.rm = TRUE), max(mnsprec2, na.rm = TRUE), length.out
= 100)
new_dta <- data.frame(bio_range)
colnames(new_dta) <- "mnsprec2"  # Ensure the column name matches the model variable

preds_means = predict(gam_model_means, newdata = new_dta, type = "response")

summary(gam_model_means)

plot(bio_range,preds_means, type="l",xlab="Precipitation seasonality
score",ylab="Proportion of clonal species",cex.lab=1.3)

# with species points

plot(bio_range,preds_means, type="l",xlab="Precipitation seasonality
score",ylab="Proportion of clonal species",cex.lab=1.3,ylim=c(-0.1,1.1))
tke = runif(length(mnsprec1)) < 0.9
points(mnsprec2[tke],jitter(mnsclon[tke],factor=0.3),pch=19,cex=0.3)
title(main=paste("R2 =",round(summary(gam_model_means)$r.sq,4)))
```

```
dev.off()



#predictions with means only, species with frequency above limitfreq only

pdf("Means-limit.pdf")

gam_model_means <- gam(mnsclon ~ s(mnsprec1,k=3),
                family = binomial, subset = mnuze)
bio_range <- seq(min(mnsprec1, na.rm = TRUE), max(mnsprec1, na.rm = TRUE), length.out = 100)
new_dta <- data.frame(bio_range)
colnames(new_dta) <- "mnsprec1"  # Ensure the column name matches the model variable

preds_means = predict(gam_model_means, newdata = new_dta, type = "response")

plot(bio_range,preds_means, type="l",xlab="Precipitation amount score",ylab="Proportion of clonal species",cex.lab=1.3)



#second component

gam_model_means <- gam(mnsclon ~ s(mnsprec2,k=3),
                family = binomial, subset = mnuze)
```

```
bio_range <- seq(min(mnsprec2, na.rm = TRUE), max(mnsprec2, na.rm = TRUE), length.out
= 100)

new_dta <- data.frame(bio_range)

colnames(new_dta) <- "mnsprec2"  # Ensure the column name matches the model variable


preds_means = predict(gam_model_means, newdata = new_dta, type = "response")


plot(bio_range,preds_means, type="l",xlab="Precipitation seasonality
score",ylab="Proportion of clonal species",cex.lab=1.3)


dev.off()



#plot habitats


pdf("Habitats-1.pdf")


for (i in 1:ncol(habitats)) {


  plot(mnscore1,mnscore2,xlab="Precipitation amount score",ylab="Precipitation
seasonality score",cex.lab=1.4,pch=19,cex=0.3)

  tke = habitats[,i] == 1

  points(mnscore1[tke],mnscore2[tke],pch=19,cex=1.2,col=plette[mnsCGO[tke]+1])

  ttle = gsub("."," ",colnames(habitats)[i],fixed=T)

  mtext("Dry",side=3,at=-.15,line=0.5,cex=0.8,adj=0.5)

  mtext("Wet",side=3,at=0.3,line=0.5,cex=0.8,adj=0.5)

  mtext("Less seasonal",side=4,at=0.2,line=0.5,cex=0.8,adj=0.5,las=3)
```

```
  mtext("Strongly seasonal",side=4,at=-0.3,line=0.5,cex=0.8,adj=0.5,las=3)

  title(main=ttle )

  legend("bottomleft", col=plette,pch=19,legend=c("nonclonal","epigeogenous rhizome","hypogeogenous rhizome", "stolon"))

}


dev.off()



#link with phylogenetic data


library(ape)

library(caper)

library(phytools)

library(geiger)


#prepare the data


tree = read.tree("Lamiaceae-Iran-GBOTB-25_1.tre")


torem = tree$tip.label[!is.na(match(sapply(tree$tip.label,function(x){strsplit(x,"_")[[1]][1]}),c("Pseudodictamnus","Moluccella","Lagochilus","Hypogomphia")))]


newtree = drop.tip(tree,torem)


tophylodata = data.frame(mnsprec1,mnsprec2,nms = names(mnsprec1),mnsclon)
```

```
sumdata = comparative.data(newtree, tophylodata, names.col = nms, vcv=FALSE,
vcv.dim=2, na.omit=FALSE,
            force.root=FALSE, warn.dropped=FALSE, scope=NULL)

#calculate phylogenetic signal in axes, two ways

xx = pgls(mnsprec1~1,lambda = "ML",data = sumdata)
summary(xx)

xx = pgls(mnsprec2~1,lambda = "ML",data = sumdata)
summary(xx)



x = sumdata$data$mnsprec1
names(x) = rownames(sumdata$data)
phylosig(sumdata$phy,x,method="lambda",test=TRUE)

x = sumdata$data$mnsprec2
names(x) = rownames(sumdata$data)
phylosig(sumdata$phy,x,method="lambda",test=TRUE)

#calculate phylogenetic signal in clonality

temptree = multi2di(sumdata$phy)
```

```
x = sumdata$data$mnsclon +1

names(x) = rownames(sumdata$data)

fitDiscrete(temptree, x, model = c("ER"), transform = c( "lambda"))

fitDiscrete(temptree, x, model = c("ARD"), transform = c( "lambda"))
```

PCA plotting code

```
plot.pca =
function(rdout,pts=T,txt=F,vars=T,pch=20,col.pt=1,cex.pt=1,cex.pt.text=1,pos.pt=4,col.sp=
1,cex.sp.names=0.8,pos.sp=4,addx=0.2,addy=0.2,
  labels=NULL,splabels=NULL,xlim=NULL,ylim=NULL,fctx=1,fcty=1,...) {
 # pts - whether to plot sites
 # txt - whether to label sites
 # pch, col.pt, cex.pt - attributes for sites (points)
 # pos.pt,labels - for site labels
 # splabels - to label species
 # col.sp,cex.sp.names,pos.sp - attributes for species (arrows and names)
 # addx,addy - constant for extension of x and y axes

 xval = scores(rdout)$species[,1] * fctx
 yval = scores(rdout)$species[,2] * fcty
 if (is.null(xlim)) {
  xl = c(min(scores(rdout)$sites[,1],xval)-addx,max(scores(rdout)$sites[,1],xval)+addx)
 } else {
```

```
  xl = xlim
 }
 if (is.null(ylim)) {
  yl = c(min(scores(rdout)$sites[,2],yval)-addy,max(scores(rdout)$sites[,2],yval)+addy)
 } else {
  yl = ylim
 }
 plot(xval,yval,xlim = xl,ylim=yl,type="p",col="white",
   xlab=paste0("Axis 1  (",100*round(summary(rdout)$cont$imp[2,1],3),"%)"),
   ylab=paste0("Axis 2
(",100*round(summary(rdout)$cont$imp[2,2],3),"%)"),cex.lab=1.4,...)
 lines(c(xl[1]-0.5,xl[2]+0.5),c(0,0),lty=2)
 lines(c(0,0),c(yl[1]-0.5,yl[2]+0.5),lty=2)
 if (is.null(splabels)) splabels = names(xval)
 if (length(pos.sp) == 1) pos.sp = ifelse(xval > 0, 4,2)
 if (vars) {
  arrows(0,0,xval,yval,lwd=2,length=0.1,col=col.sp,...)
  text(xval,yval,splabels,pos=pos.sp,cex=cex.sp.names,col=col.sp,...)
 }
 if (pts)
  points(scores(rdout)$sites[,1],scores(rdout)$sites[,2],pch=pch,col=col.pt,cex=cex.pt,...)
 if (length(pos.pt) == 1)
  if (pts) pos = pos.pt else pos = NULL else pos = pos.pt
 if (is.null(labels)) labels=rownames(scores(rdout)$sites)
 if (txt)
```

```
text(scores(rdout)$sites[,1],scores(rdout)$sites[,2],labels=labels,col=col.pt,cex=cex.pt.tex
t,pos=pos,...)

}
```

Bar-Plot code

```
# Load necessary libraries

library(readxl)

library(dplyr)

library(ggplot2)

library(tidyr)


# Set the file path

file_path <- "G:/My Drive/Paper/1- Bar-Plot/finilize excel-1.xlsx"


# Read the Excel file

data <- read_excel(file_path)


# List of habitat columns to analyze

habitat_columns <- c("Desert Steppes", "Semi-desert Steppes", "Chasmophytic
Vegetation",

            "Montane Steppe Shrublands", "Juniperus Woodlands",

            "Forests", "Wetlands")

proportions <- data %>%

  select(Species, Dominant_CGO, all_of(habitat_columns)) %>%
```

```
 pivot_longer(cols = all_of(habitat_columns), names_to = "Habitat", values_to =
"Presence") %>%
 filter(Presence == 1) %>%
 group_by(Habitat, Dominant_CGO) %>%
 summarise(Species_Count = n(), .groups = "drop") %>%
 group_by(Habitat) %>%
 mutate(Proportion = Species_Count / sum(Species_Count)) %>%
 mutate(
  Habitat = factor(Habitat,
           levels = c("Desert Steppes", "Semi-desert Steppes", "Chasmophytic Vegetation",
                "Montane Steppe Shrublands", "Juniperus Woodlands", "Forests",
"Wetlands")),
  # set the order for stacking
  Dominant_CGO = factor(Dominant_CGO,
              levels = c("main root", "hypogeogenous rhizome", "epigeogenous rhizome",
"stolon" ))
 )


png("G:/My Drive/Paper/1- Bar-Plot/stacked_bar.png", width = 1200, height = 800, res = 150)



# Stacked Bar Chart with Custom Colors
ggplot(proportions, aes(x = Habitat, y = Proportion, fill = Dominant_CGO)) +
 geom_bar(stat = "identity") +
 labs(title = "Contribution of belowground growth forms to biomes",
    x = "Habitat", y = "Proportion", fill = "Belowground Growth Forms") +
```

```
  scale_fill_manual(values = c(
    "hypogeogenous rhizome" = "#56B4E9",  # Okabe–Ito Yellow
    "epigeogenous rhizome"  = "#D55E00",  # Okabe–Ito Bluish green
    "main root"             = "#003366",  # Okabe–Ito Blue
    "stolon"                = "#F0E442"   # Okabe–Ito Reddish purple
  )) +
  theme_minimal()


dev.off()
```
