# Supplementary figures and images for: Plants clonal strategies are well associated with aridity gradients: insights from Lamiaceae family in the SW and Central Asia
Source: AoB Plants. 2025 Dec 8;17(6):plaf069. doi: 10.1093/aobpla/plaf069 (PMC12723225; doi:10.1093/aobpla/plaf069)

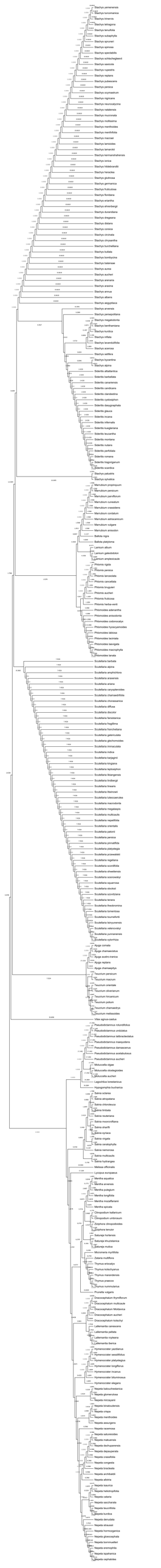

Supplement: plaf069_Supplementary_Data [file plaf069_supplementary_data.zip › Figure S1.pdf]
